# Antifibrotic treatment response and prognostic predictors in patients with idiopathic pulmonary fibrosis and exposed to occupational dust

**DOI:** 10.1186/s12890-019-0930-7

**Published:** 2019-09-05

**Authors:** Valeria Casillo, Stefania Cerri, Andrea Ciervo, Mariarita Stendardo, Lamberto Manzoli, Maria Elena Flacco, Maurizio Manno, Marialuisa Bocchino, Fabrizio Luppi, Piera Boschetto

**Affiliations:** 1 Department of Medical Sciences, University of Ferrara, Via Fossato di Mortara 64, Ferrara, Italy; 2 Center for Rare Lung Diseases, University Hospital of Modena, Via del Pozzo 71, Modena, Italy; 3 Section of Occupational Medicine, Department of Public Health, University of Naples Federico II, Via Sergio Pansini 5, Naples, Italy; 4 Department of Clinical Medicine and Surgery, Section of Respiratory Diseases, University of Naples Federico II, Via Sergio Pansini 5, Naples, Italy; 5 School of Medicine and Surgery, University of Milano-Bicocca, Piazza dell’Ateneo Nuovo 1, Milan, Italy

**Keywords:** Idiopathic pulmonary fibrosis, Occupational dust, Pirfenidone, Nintedanib, Prognosis, GAP

## Abstract

**Background:**

Idiopathic Pulmonary Fibrosis (IPF) is an aggressive interstitial lung disease with an unpredictable course. Occupational dust exposure may contribute to IPF onset, but its impact on antifibrotic treatment and disease prognosis is still unknown. We evaluated clinical characteristics, respiratory function and prognostic predictors at diagnosis and at 12 month treatment of pirfenidone or nintedanib in IPF patients according to occupational dust exposure.

**Methods:**

A total of 115 IPF patients were recruited. At diagnosis, we collected demographic, clinical characteristics, occupational history. Pulmonary function tests were performed and two prognostic indices [Gender, Age, Physiology (GAP) and Composite Physiologic Index (CPI)] calculated, both at diagnosis and after the 12 month treatment. The date of long-term oxygen therapy (LTOT) initiation was recorded during the entire follow-up (mean = 37.85, range 12–60 months).

**Results:**

At baseline, patients exposed to occupational dust [≥ 10 years (*n* = 62)] showed a lower percentage of graduates (19.3% vs 54.7%; *p* = 0.04) and a higher percentage of asbestos exposure (46.8% vs 18.9%; p 0.002) than patients not exposed [< 10 years (*n* = 53)]. Both at diagnosis and after 12 months of antifibrotics, no significant differences for respiratory function and prognostic predictors were found. The multivariate analysis confirmed that occupational dust exposure did not affect neither FVC and DLCO after 12 month therapy nor the timing of LTOT initiation.

**Conclusion:**

Occupational dust exposure lasting 10 years or more does not seem to influence the therapeutic effects of antifibrotics and the prognostic predictors in patients with IPF.

**Electronic supplementary material:**

The online version of this article (10.1186/s12890-019-0930-7) contains supplementary material, which is available to authorized users.

## Background

Idiopathic pulmonary fibrosis (IPF) is a chronic, progressive, fibrosing interstitial pneumonia of unknown cause defined by the histopathologic and/or radiologic pattern of usual interstitial pneumonia (UIP) [1]. It appears mainly in older adults and is associated with unrelenting decline in lung function, advancing respiratory failure and high mortality rate.

The possible involvement of occupational exposure (past and/or still going) has been considered and investigated with regards both to the pathogenesis and the progression of the disease. Previous observational studies have implicated occupational exposures to metal, stone and wood dust, chemical fumes and organic dust in the pathogenesis of IPF and subjects exposed to these have an increased risk of developing the condition [2, 3]. Although a causal relationship between occupational exposure and IPF has not been recognized yet, the epidemiological evidence of occupations proved to be related to IPF is continuously growing [4]. Notably, males with a history of heavy smoking and occupational exposure to harmful agents, specifically birds and wood dust, have been identified as a particular group at increased risk of developing severe pulmonary fibrosis [5].

While the involvement of occupational and environmental factors in the pathogenesis of IPF have been explored to a considerable extent, studies investigating possible influence of job activity on the clinical features and prognosis of this condition are few. Indeed, only one report evaluated the clinical, physiologic and radiologic characteristics and the prognosis in patients with IPF according to their occupation. The main finding was that dust-exposure occupation was significantly associated with mortality after adjusting for major confounders such as age, sex, lung function parameters and radiologic aspects [6]. The little data available on the possible impact of environmental factors on IPF prognosis shows that both long-term exposure to and average concentration of ambient particulate (PM_10_ and PM_2.5_) are associated with overall mortality and an increase in the rate of decline of forced vital capacity (FVC) in patients with IPF [7, 8].

Investigations taking into account occupational and environmental exposure in the assessment of the response to antifibrotic therapy (pirfenidone and nintedanib) in IPF patients are lacking.

Therefore, in this study we evaluated clinical and functional characteristics at diagnosis and after 12 month treatment in patients with IPF according to their occupational dust exposure. In addition, we investigated if such occupational exposure might influence the prognosis of IPF in terms of mortality and initiation to long-term oxygen therapy (LTOT). In fact, although the latter can vary from one physician to another, it indicates an undoubted important worsening of the condition.

## Methods

### Study design and subjects

From January 2014 to December 2018 an observational prospective cohort study was carried out. We enrolled all consecutive patients (*n* = 115) referred to the Center for rare lung disease of the University Hospital of Modena and to the Pneumological clinic of the Federico II University Hospital of Napoli with a new diagnosis of IPF. Diagnosis of IPF was performed according to the 2011 criteria of the American Thoracic Society/European Respiratory Society [1].

Data were registered in an ad hoc database. At baseline, we collected for each patient socio-demographic characteristics [age, sex, smoking status, amount smoked (pack-years), educational level, lifetime occupational history, exposure to asbestos] and clinical parameters [body mass index (BMI), dyspnea, time of onset of respiratory symptoms, comorbidities, pharmacological treatment, disability perceived in relation to health conditions and psychological distress]. We also recorded pulmonary function tests (PFTs) and calculated the prognostic indices Gender, Age, Physiology (GAP) and the Composite Physiologic Index (CPI).

Of the 115 patients recruited, 101 were treated with pirfenidone or nintedanib and of those 89 agreed to perform PFTs after 12 months of therapy. The study was concluded when the last recruited patient completed the 12 month treatment period.

All the 115 patients were censored for death and the date of long-term oxygen therapy (LTOT) initiation for the entire study period.

A written informed consent was provided by all participants before recruitment. The study was conducted in accordance to the Declaration of Helsinki and approved by the institutional ethics committees of University Hospitals of Ferrara, Modena and Napoli (N.160494).

### Measurements

#### Sociodemographic and clinical features

BMI was calculated by dividing weight (Kg) by height squared (m^2^). The number of pack years was figured as number of cigarettes smoked per day x number of years smoked/20. Level of dyspnea was assessed by the modified Medical Research Council (mMRC) scale. Disability perceived in relation to health conditions and psychological distress were measured using the World Health Organization Disability Assessment Schedule (WHODAS) 12 items version [9] and the Hospital Anxiety and Depression Scale (HADS) [10], respectively.

#### Occupational exposure

We collected a complete occupational history, including a job activity checklist and a specific checklist of occupational dust exposure related to IPF (organic dusts; stone, sand, or metal dusts; and wood dust) [6].

The recorded information included job title, tasks performed, detailed description of the activity, use of individual protection devices, substances contact, years spent in each job and in each occupational dust exposure. Occupational exposure was defined as occupational exposure to dusts related to IPF by 10 or more years before diagnosis [5].

#### Lung function

PFTs were performed according to international criteria [11]. To assess the possible influence of occupational exposure on respiratory function and disease progression, forced vital capacity percent of predicted (FVC % pred.) and diffusing capacity of the lungs for carbon monoxide percent of predicted (DLCO % pred.) at diagnosis and at 12 month follow-up were used.

#### Prognostic predictors

Gender, Age, Physiology (GAP) index is a validated, multidimensional tool that predicts mortality in IPF. The calculation of the score encompasses gender (G), age (A) and two lung physiology variables (P) (FVC % pred. and DLCO % pred.). Points are assigned for each variable to obtain a total range from 0 to 8. According to this score, patients are classified in stage I (0–3 points), stage II (4–5 points), or stage III (6–8 points) [12]. As the GAP increases, the probability of mortality rises.

Composite Physiologic Index (CPI) is a validated, multidimensional index that correlates with the extent of pulmonary fibrosis and mortality and thus predicts IPF progression [13, 14]. CPI is computed as follows: CPI = 91.0 - (0.65 × DLCO % predicted) - (0.53× FVC % predicted) + (0.34 × FEV_1_% predicted). Higher CPI scores indicate more severe fibrosis and poorer prognosis [14].

Both GAP and CPI index were calculated at diagnosis and at 12 month follow-up, although GAP has not been circumstantially validated at 1 year.

#### Initiation of long-term oxygen therapy (LTOT)

LTOT can be defined as oxygen used for at least 15 h per day in chronically hypoxaemic patients [15]. In patients with IPF, LTOT initiation may be a marker of poor prognosis as it predicts a median survival of less than 18 months [13]. The date of long term oxygen initiation was recorded for each participant.

### Data analysis

IPF patients were classified according to exposure in two groups: exposed (≥ 10 years) and not exposed (< 10 years).

First, at baseline, we investigated whether the two groups differed in selected demographic variables, clinical characteristics and lung functional parameters (FVC; FEV_1_; DLCO; GAP and CPI index), using chi-squared and Kruskal-Wallis tests for categorical and continuous variables, respectively.

Second, we evaluated whether the two groups of patients differed in lung function measurements and prognostic predictors measured after 12 month therapy.

Overall, a total of eight multiple regression models were fit. However, as FVC and FEV1 at diagnosis were highly collinear (Spearman rho = 0.96), only the analyses related to FVC were reported to avoid redundancy.

In all models, covariates were included in a stepwise forward process using the following criteria: clinical relevance, with gender, age at symptoms onset, smoke and occupational dust exposure forced to entry. Occupational dust exposure was treated either as continuous or ordinal variable, including the above mentioned two groups of exposure (< 10 years and ≥ 10 years) as dummy variables.

The validity of final regression models was assessed as follows: the assumption of constant error variance was checked graphically, plotting Pearson residuals vs. fitted values, and formally, using the Cook-Weisberg test for heteroskedasticity. High leverage observations were identified by computing Pearson, standardized and studentized residuals, and Cook’s D influence. In all models, we found less than 10 high-leverage observations, excluding which we noted no substantial changes.

As a separate, additional evaluation, we tested with Cox proportional hazard analysis whether there was any evidence that starting oxygen therapy depended on: (a) previous work exposure to dusts lasting ≥10 years; (b) number of cigarette pack-years; (c) baseline FEV1; (d) IPF stage at baseline (separately assessed using GAP and CPI index). We selected all covariates a priori, and, in order to avoid overfitting, we fitted two separate models, each including one of the two IPF scoring systems, with all other covariates remaining stable. Finally, we used Schoenfeld’s test to check the validity of proportional hazards assumption for both models.

Statistical significance was defined as a two-sided *p*-value< 0.05, and all analyses were carried out using Stata, version 13.1 (Stata Corp., College Station, Texas, USA, 2013).

## Results

### Baseline characteristics of the study population

Table 1 summarizes the baseline characteristics of the 115 study patients according to occupational exposure. The mean duration of dust exposure related to IPF was 36.74 ± 13.75 and 1.2 ± 2.8 years in the exposed and not exposed group, respectively. The majority of subjects were male and current or former smokers with a similar number of pack/years in the two groups. There was no significant difference in age, age at respiratory symptoms onset, the percentage of patients with family history of IPF and with the distribution of the most common self-reported comorbidities between IPF subjects with and without occupational exposure. Likewise, dyspnea, classified according to the mMRC dyspnea score, the number of subjects who did not start IPF treatment, those given pirfenidone and those treated with nintedanib were similar in the two groups.

**Table 1:** Baseline demographic and clinical characteristics of the study population according to occupational dust exposure

Variables^a^	Exposed (*n* = 62)	Not exposed (*n* = 53)	*p*
Male gender, %	79.0	69.8	0.21
Mean age, years (SD)	69.6 (7.3)	68.7 (8.7)	
Mean age at symptoms onset (SD)	68.2 (7.9)	67.7 (9.1)	
Mean time-span between symptoms onset and diagnosis, months (SD)	15.7 (17.3)	17.8 (25.0)	
Educational level, %			
-Primary/secondary school	50.1	28.3	
-High school	30.6	17.0	
-University degree	19.3	54.7	**0.04**
Mean BMI, kg/m^2^ (SD)	28.0 (4.3)	28.1 (4.1)	
Smoking status:			
-Cigarette smoke (current or former), %	75.8	67.9	
-Mean Pack/year (SD)	25.6 (24.0)	24.9 (27.8)	
Comorbidities %			
-None	11.3	3.8	
-GERD	24.2	11.3	
-Cancer	6.5	13.2	
-Respiratory diseases	9.6	15.1	
-Cardiovascular diseases	40.3	30.1	
-Others	9.1	26.5	
Family history of IPF, %	9.7	9.4	
mMRC Breathlessness Scale grade^b^, %			
-Grade 0	4.8	9.3	
-Grade 1	21.0	18.9	
-Grade 2	32.3	43.4	
-Grade 3	29.0	22.6	
-Grade 4	12.9	5.7	
Exposure to asbestos, %	46.8	18.9	**0.002**
Mean duration of asbestos exposure, years (SD)	8.9 (12.4)	5.8 (13.6)	
Mean WHODAS 2.0 score (SD)	24.7 (18.6)	20.8 (19.2)	
Mean HADS-A score (SD)	5.9 (3,2)	6.2 (3.5)	
Mean HADS-D score (SD)	7.0 (3.1)	5.6 (3.4)	
Pharmacological treatment, %			
-None	16.1	7.6	
-Pirfenidone	46.8	50.9	
-Nintedanib	37.1	41.5	

Exposed = occupational exposure to dust ≥10 years

Not exposed = occupational exposure to dust < 10 years

*SD* Standard deviation, *BMI* Body Mass Index, *GERD* Gastro-esophageal reflux disease, *mMRC* modified Medical Research Council, *WHODAS 2.0* WHO Disability Assessment Schedule 2.0 tool, *HADS-A* Hospital Anxiety and Depression Scale-Anxiety domain, *HADS-D* Hospital Anxiety and Depression Scale-Depression domain

^a^Chi-squared test for categorical variables; Kruskal-Wallis test for normally-distributed and non normally distributed continuous variables, respectively. *P*-values that are not reported were > 0.05. *P*-values < 0.05 are reported in bold

^b^Each grade of the mMRC Breathlessness Scale was scored as follows: Grade 0 = Not troubled by breathlessness except on strenuous exercise; Grade 1 = Short of breath when hurrying on the level or walking up a slight hill; Grade 2 = Walks slower than most people on the level, stops after a mile or so, or stops after 15 min walking at own pace; Grade 3 = Stops for breath after walking about 100 yards or after a few minutes on level ground; Grade 4 = Too breathless to leave the house, or breathless when undressing

The exposed group counted a lower percentage of patients with an academic degree and a higher percentage who reported asbestos exposure.

### Pulmonary function tests and prognostic indices

No significant imbalance for pulmonary function parameters, including FVC % of the predicted and DLCO % of the predicted, was found between the two groups both at baseline and after 12 month therapy assessment. Similarly, prognosis evaluated through GAP graded score and CPI index was comparable in patients with and without occupational exposure (Table 2, data are restricted to 89 subjects who underwent pharmacological therapy and spirometry at diagnosis and 12 month treatment visit). Dividing the patients into three clinically meaningful groups according to change in FVC after therapy ≥10%, < 10% and stable/increased, we did not observe appreciable differences with regards to neither the occupational exposure nor the clinical or demographic features [see Additional file 1].

**Table 2:** Pulmonary function parameters and prognosis indices according to dust exposure at baseline and 12 month treatment

Variables^a^	Exposed (*n* = 46)^b^		Not exposed (*n* = 43)^b^	
	Baseline assessment	12-month assessment	Baseline assessment	12-month assessment
Mean FEV_1_, L (SD)	2.2 (0.5)	2.1 (0.5)	2.1 (0.6)	2.1 (0.6)
Mean FEV_1_, % predicted (SD)	89.9 (21.2)	86.8 (19.9)	84.3 (20.3)	80.8 (19.2)
Mean FVC, L (SD)	2.6 (0.6)	2.5 (0.6)	2.5 (0.8)	2.4 (0.8)
Mean FVC, % predicted (SD)	84.0 (19.7)	80.5 (17.8)	76.7 (18.5)	73.7 (18.6)
Mean DLCO, % predicted (SD)	49.4 (14.7)	44.8 (18.0)	47.7 (12.2)	45.6 (13.3)
Mean TLC, L (SD)	4.5 (1.1)	4.3 (1.1)	4.2 (1.2)	4.1 (1.1)
Mean TLC, % predicted (SD)	76.1 (16.9)	71.4 (15.7)	71.8 (14.6)	67.6 (15.9)
GAP score, %				
- 0–3 (IPF stage I)	40.9	31.8	40.5	36.6
- 4–5 (IPF stage II)	47.7	47.7	52.4	46.3
- 6–8 (IPF stage III)	11.4	20.5	7.1	17.1
Mean GAP score (SD)	3.8 (1.3)	4.0 (1.5)	3.7 (1.2)	4.0 (1.5)
Mean IPF stage (SD)	1.7 (0.7)	1.9 (0.7)	1.7 (0.6)	1.8 (0.7)
Mean CPI score (SD)	44.9 (12.3)	50.1 (15.1)	47.4 (9.9)	49.1 (11.1)

Exposed = occupational exposure to dust ≥10 years

Not exposed = occupational exposure to dust < 10 years

*SD* Standard deviation, *FEV*_*1*_ Forced Expiratory Volume at 1st second, *FVC* Forced Vital Capacity, *DLCO* Diffusing capacity of the lungs for Carbon Monoxide, *TLC* Total Lung Capacity, *GAP* Gender, Age and Physiology, *CPI* Composite Physiologic Index

^a^Chi-squared test for categorical variables; Kruskal-Wallis test for normally-distributed and non-normally distributed continuous variables, respectively. As all *p*-values were > 0.05 they were not reported to avoid redundancy

^b^Analysis restricted to 89 subjects in pharmacological treatment and with complete respiratory function tests at diagnosis and after 12 months of therapy

Table 3 shows the results of a multivariate regression model for factors significantly related to FVC and DLCO measured at 12 month treatment, after adjusting for confounders. Male gender and 1 point increase of FVC % pred. at baseline were significantly associated with an increase of FVC % pred. After 12 months of treatment (*p* < 0.001). Likewise, 1 point increase of DLCO % pred. at baseline was related to an increase of DLCO % pred. After 1 year of therapy (*p* < 0.001). Conversely, occupational exposure did not affect neither FVC nor DLCO.

**Table 3:** Relationship between baseline characteristics of the population and FVC and DLCO at 12 month treatment

Variables	FVC% pred. 1-year after treatment		DLCO% pred. 1-year after treatment	
	Adjusted coeff. (95% CI)	*p*	Adjusted coeff. (95% CI)	*p*
Mean age at symptoms onset, 10-year increase	−0.04 (− 0.12; 0.03)	0.2	0.17 (− 0.08; 0.43)	0.2
Male gender	0.44 (0.25; 0.63)	**< 0.001**	3.11 (−3.45; 9.69)	0.3
Mean BMI, 10-point increase	−0.08 (− 0.25; 0.09)	0.3	0.04 (− 0.52; 0.61)	0.9
Smoking status:				
-Cigarette smoke, yes vs no	0.13 (−0.01; 0.27)	0.07	−1.34 (−5.62; 2.94)	0.5
-Mean pack/year, 10-unit increase	−0.01 (− 0.04; 0.02)	0.5	− 0.15 (− 1.17; 0.88)	0.8
History of GERD, yes vs no	0.09 (− 0.06; 0.26)	0.3	0.98 (−4.61; 6.58)	0.7
Familiar history of IPF, yes vs no	0.00 (−0.21; 0.20)	0.9	2.75 (−3.84; 9.34)	0.4
Exposed vs not exposed^a^	0.06 (−0.06; 0.19)	0.3	1.17 (−3.01; 5.17)	0.6
Mean duration of exposure, 10-y increase	−0.00 (− 0.03; 0.03)	0.9	− 0.01 (− 0.12; 0.09)	0.8
Asbestos exposure, yes vs no	− 0.04 (− 0.17; 0.09)	0.6	−2.76 (− 7.25; 1.73)	0.2
FVC % predicted, 1% increase				
-Baseline FVC	0.01 (0.01; 0.02)	**< 0.001**	−1.96 (−6.47; 2.54)	0.4
-Pre-therapy FVC	0.02 (0.01; 0.02)	**< 0.001**	−0.09 (− 0.23; 0.05)	0.2
DLCO % predicted, 1% increase				
-Baseline DLCO	0.00 (−0.01; 0.01)	0.3	0.73 (0.52; 0.94)	**< 0.001**
-Pre-therapy DLCO	0.00 (−0.00; 0.01)	0.5	0.71 (0.51; 0.91)	**< 0.001**
mMRC Breathlessness Scale grade, 1-grade increase	−0.01 (− 0.08; 0.06)	0.7	−1.31 (−3.57; − 0.95)	0.3

Analysis restricted to 89 subjects in pharmacological treatment and with complete respiratory function tests at diagnosis and after 12 month of therapy. *P*-values < 0.05 are reported in bold

*FVC* Forced Vital Capacity, *DLCO* Diffusing capacity of the lungs for Carbon Monoxide, *CI* Confidence Interval, *coeff*. coefficient, *BMI* Body Mass Index, *GERD* Gastro-esophageal reflux disease, *mMRC* modified Medical Research Council

^a^Exposed = occupational exposure to dust ≥10 years, not exposed = occupational exposure to dust < 10 years

### Initiation of long-term oxygen therapy (LTOT) and deaths

The mean follow-up was 37.85 (range 12–60) months. Throughout this period 38 of the 115 patients (33%) started LTOT. Comparing these subjects to those who did not need LTOT, no significant difference for occupational dust exposure was found (57.9% vs 52%; with a mean duration of exposure of 23.7 ± 22.4 vs 18.8 ± 19.4, respectively). Patients receiving LTOT had a higher baseline disability score (33.1 ± 21.1 vs 17.9 ± 15.5, *p* < 0.001), lower pulmonary function values (FEV_1_, FVC, TLC and DLCO *p* < 0.002 for all) and poor prognosis, as indicated by a higher GAP (4.2 ± 1.4 vs 3.6 ± 1.3, *p =* 0.02) and CPI index (52.0 ± 12.4 vs 43.2 ± 11.0, *p* < 0.001) calculated at baseline. Using a Cox proportional hazard model, we explored potential predictors of LTOT initiation, in particular: work exposure to dusts, cigarette pack/years, and baseline FEV_1_, GAP and CPI index. Results are shown in Table 4. Increasing FEV_1_ (L) reduces the risk to undergo LTOT, whereas a higher GAP and CPI index are predictive of LTOT initiation. Occupational dust exposure was confirmed to be unrelated to LTOT.

**Table 4:** Potential predictors of starting long term oxygen therapy

Variables	Oxygen therapy HR (95% CI)	*p*
Exposed vs not exposed^a^	0.63 (0.28–1.40)	0.3
Mean pack/year, 10-unit increase	1.17 (0.98–1.39)	0.09
FEV_1_, L 1-unit increase	0.38 (0.17–0.85)	**0.020**
GAP score, 1-level increase	1.89 (1.30–2.76)	**0.001**
CPI score, 1-level increase	1.07 (1.02–1.12)	0.008

*HR* Hazard Ratio, *CI* Confidence Interval 95%, *FEV*_*1*_ Forced Expiratory Volume at 1st second, *GAP* Gender, Age and Physiology, *CPI* Composite Physiologic Index. *P*-values < 0.05 are reported in bold.

^a^Exposed = occupational exposure to dust ≥10 years, not exposed = occupational exposure to dust < 10 years

The number of deaths was too small (*n* = 21, 18.2%) to allow a proper statistical analysis. Patients died for progression or exacerbation of IPF (*n* = 11), cardiovascular complications (*n* = 4), multiple organ failure (*n* = 3) and cancer (*n* = 1). The cause of death was unknown for 2 patients due to lack of information.

## Discussion

In this study, we have shown that 62 of the 115 patients (54%) with a diagnosis of IPF had an occupational dust exposure, defined as lasting 10 or more years. IPF patients with occupational dust exposure exhibited a lower educational level and a higher frequency of reported asbestos exposure. Age, gender, smoking history, dyspnea, comorbidities, lung function parameters and prognostic indices (GAP, CPI) were similar in patients with and without occupational exposure to dusts, both at diagnosis and after 12 month therapy with pirfenidone or nintedanib. Occupational dust exposure did not seem to influence the timing of initiation of LTOT in the follow-up period.

The finding of a lower educational level in the dust-exposed group was expected and corresponds to the results of other studies comparing the educational level between workers exposed to various dusts and administration office workers [16].

Also the higher percentage of IPF patients exposed to asbestos in the dust-exposed group was not surprising. Indeed, these two occupational exposures are often associated with a number of work activities as carpentry, engineering, construction and quarrying. In our study population, asbestos exposure did not seem to affect both clinical characteristics and lung function measurements either before and after 12 months of IPF treatment, indicating a careful exclusion of an asbestos etiology in the diagnostic workup [17]. It is noteworthy that the differential diagnosis between asbestosis and IPF is challenging, and an accurate patient recall of historic asbestos exposure is an essential factor in discriminating the two conditions. In our study, of almost 50% of patients in the exposed group who had exposure to asbestos, 17 had an occupational exposure, while the others were exposed non-occupationally (household and neighbourhood); of the non-exposed group only 3 had been exposed in occupational settings. The estimated non-occupational exposure was not sufficient to support a diagnosis of asbestosis. Particular attention was paid in investigating not only the timing but also the intensity of asbestos exposure as a heavy exposure for few years may be equivalent to a small exposure for many years. Considering time and intensity of exposure, the latter calculated approximately by job title and working history, only a few patients in the exposed group had an asbestos exposure that could support a diagnosis of asbestosis. As patients suffering from asbestosis experience a better survival rate than the general IPF population, we took specific care to ascertain a correct differential diagnosis. In subjects with a significant asbestos exposure, IPF were diagnosed during a multidisciplinary dynamic discussion after formal work-up, including extensive patient history, chest radiograph and CT scan (also looking for benign asbestos-associated pleural disease such as pleural plaques, diffuse pleural fibrosis and benign asbestos pleural effusion), but rarely searching for asbestos bodies in BAL and lung tissue.

At the diagnosis, age, age at symptoms onset and lung function parameters, particularly FVC % and DLCO % predicted, were found similar in patients exposed and not exposed. These findings seem to disagree with those of Lee et al. [6] who found an early onset of IPF and a reduced DLCO in dust-exposed workers. However, they evaluated occupational exposure differently: 1) medical and occupation records were collected retrospectively from a web-based registry; 2) patients were categorized into five groups according to occupations (unemployed or homemakers; farmers, fishers or ranchers; sales or service personnel; clerical or professional personnel; and workers exposed to dusts, but excluding organic dusts); 3) an early onset of IPF and a reduced DLCO were found significant when dust-exposed workers were compared to unemployed or homemakers but to none of the other group, making this result rather weak.

As reported above, our patients exposed to occupational dust did not exhibit reduction in lung function parameters at the IPF diagnosis compared to those non exposed. In population based studies, the issue of a possible effect of dust exposure at work on long-term excess decline in lung function is still controversial. Indeed, the results of previous reports are inconsistent and the differences in study design, qualitative and quantitative dust exposure and the lung function indices chosen as outcome (FEV_1_, FVC, and/or FEV_1_/FVC) make them even less conclusive. Lastly, smoking is a major injury for lung function and, therefore, a crucial bias in the evaluation of the lung effects of occupational dust exposure [18].

Both pirfenidone and nintendanib have been shown to lessen the decline in pulmonary function in patients with IPF [19]. In particular, they reduce the number of patients experiencing a decline in FVC of 10% or greater with the result of slowing the disease progression, as compared to no therapy [19]. Although we could not compare the rate of disease progression between patients with and without pharmacological treatment, we found that exposure to dust at work does not seem to impact on the beneficial effect of 1 year pirfenidone and nintendanib. In fact, the stability of lung function after 1 year of therapy was present in patients with and without occupational dust exposure, and dust exposure was not associated with post-treatment FVC % pred. and DLCO % pred. Furthermore, the percentage of patients with occupational dust exposure did not differ between those who exhibited a decrease ≥10%, a decrease < 10% and a stability or increase in pre-post therapy FVC % pred. To our knowledge, this is the first study investigating the possible influence of occupational exposure to dusts on IPF therapy, and it suggests that the beneficial effect of pirfenidone and nintendanib on lung function is not influenced by the exposure to occupational dust.

In the present IPF patients, no difference was found in GAP index score for IPF mortality, CPI and the percentage of those who initiated LTOT based on dust exposure at work. Whether occupational dust exposure could have an impact on the prognosis of IPF patients is still unknown. Lee et al. reported that exposure to dust was associated with an increased risk of mortality in patients with IPF in a survival analysis where occupation was adjusted for age, sex, pulmonary function, arterial partial pressure of oxygen (PaO_2_), and honeycombing on the HRCT scan, but not in a survival analysis where occupation was adjusted for GAP stage [6], indicating that the question of a possible association between occupational dust exposure and IPF prognosis remains unanswered.

In the present study also CPI was not related to dust exposure, strengthening the result of GAP index score. Interestingly, the relationship between CPI and occupational exposure in patients with IPF has not been previously investigated.

IPF patients who started LTOT during follow-up showed lower levels of respiratory function (FEV_1_; FVC; DLCO; TLC), worse prognostic indices (CPI; GAP) and a higher level of disability at diagnosis compared to patients who did not start LTOT. Increased GAP score and CPI at diagnosis were potential predictors of long-term oxygen therapy initiation, whereas a higher level of FEV_1_ was protective. Occupational exposure did not affect LTOT. With regards to respiratory function parameters, our data confirm the results of previous studies that found an association between LTOT and low levels of FEV_1_, FVC and DLCO in patients with pulmonary fibrosis and other chronic lung diseases [20, 21].

This observational study has some limitations. First, occupational history was self-reported and, therefore, potentially involving a recall bias. Data on measurements of intensity of dust exposure at work, as well as the latency between exposure cessation and disease diagnosis, were not available. However, years of exposure were gathered and the average of 36.74 years proves a considerable exposure. Furthermore, as the intensity of dust exposure generally differs between different occupations, we divided our dust exposed group in two categories based on data collected on job title: highest/heaviest dust exposure and moderate dust exposure. Of the 46 exposed, 50% had probable intense exposure (for example: turners, construction workers, carpenters, textile workers) and the remaining 50% moderate exposure (example: cleaners, farmers, breeders). We did not find any significant difference in spirometric parameters at diagnosis and at 12 months as well as in the number of deaths and in the timing of initiation of LTOT between the two categories of the exposed and between the highest/heaviest dust exposed compared with the non-exposed (data not shown).

Second, complete data on lung function at diagnosis and after 12 months were available for only 89 patients, all under pharmacological treatment. Thus, given the absence of follow-up data for the group of untreated patients, it was not possible to comment on whether anti-fibrotic treatment is effective in slowing disease progression/reduce the number of patients experiencing decline in FVC > 10% regardless of dust exposure. Similarly, there are no data to demonstrate that dust exposure does not impact on the beneficial effect of anti-fibrotic therapy at 1 year.

Third, the sample size of the study population was relatively small to evaluate the exposure effect of each occupational dust, i.e. organic dust, stone, sand, metal and wood dust. Although this might have implicated an underestimation of a specific kind of dust, we believe that the investigation on occupational dust exposure in its whole is particularly informative of the possible impact of occupations on IPF prognostic predictors and antifibrotic treatment. The limited sample size was also responsible, at least in part, for the small number of deaths we registered during the follow-up period and, therefore, for the missed opportunity to perform a mortality analysis. Nevertheless, we examined two well-recognized prognostic indices (GAP and CPI) and the initiation of LTOT, another robust prognostic predictor, contributing to address the question of the relationship, if any, between occupational dust exposure and IPF prognosis. It should be noted that available literature on this issue at present is scarce and controversial [6, 22]. Finally, it has to be mentioned that GAP has been validated at diagnosis and not at 1 year. Nevertheless, we thought we could calculate and report it since it has been found that GAP models performed similarly in diagnosis and in pooled follow-up visits [12].

## Conclusions

IPF patients with occupational dust exposure have clinical and functional characteristics similar to non-exposed IPF patients at diagnosis. Also the effect of 12 month antifibrotic therapy was found analogous in the two groups, irrespective of dust exposure. The prognostic indices, GAP and CPI, and the timing of LTOT initiation did not appear to be affected by such occupational exposure. Although the results of our study were mainly negative, we believe that it is worthy to explore each possible factor which may influence the natural history and the response to therapy of IPF, a disease associated to rapid progression, fast worsening quality of life and high mortality.

## Additional file

Additional file 1: Characteristics of population according to change in FVC from baseline to 12 month treatment, (DOC 60 kb)

## Data Availability

The datasets used and/or analyzed are available from corresponding author upon reasonable request.

## Abbreviations

BMI: Body mass index
CPI: Composite physiologic index
DLCO: Diffusing capacity of the lungs for carbon monoxide
FEV_1_: Forced expiratory volume in the 1st second
FVC: Forced vital capacity
GAP: Gender, age, physiology
HADS: Hospital anxiety and depression scale
IPF: Idiopathic pulmonary fibrosis
LTOT: Long-term oxygen therapy
mMRC: modified Medical Research Council
PFTs: Pulmonary function tests
UIP: Usual interstitial pneumonia
WHODAS: World Health Organization disability assessment schedule
