# Supplementary material for: Antifibrotic treatment response and prognostic predictors in patients with idiopathic pulmonary fibrosis and exposed to occupational dust
Source: BMC Pulm Med. 2019 Sep 5;19:170. doi: 10.1186/s12890-019-0930-7 (PMC6727559; doi:10.1186/s12890-019-0930-7)
Supplement: Supplementary file 1 — Characteristics of population according to change in FVC from baseline to 12 month treatment, (DOC 60 kb) [file 12890_2019_930_MOESM1_ESM.doc]

**Additional file 1. Characteristics of population according to change in FVC from baseline to 12 month treatment**

| *Variables** | **FVC% pred.**  **decrease ≥10%**  **(n=21)°** | **FVC% pred.**  **decrease <10%**  **(n=29)°** | **FVC% pred.**  **stable or increased**  **(n=39)°** |
| --- | --- | --- | --- |
|
| **Male gender, %** | 71.4 | 75.9 | 76.9 |
| **Mean age at baseline, years (SD)** | 68.3 (8.1) | 69.5 (7.1) | 68.7 (8.2) |
| **Mean age at symptoms onset, years (SD)** | 68.1 (6.2) | 68.5 (7.6) | 66.6 (9.8) |
| **Mean time-span between symptoms onset and diagnosis, months (SD)** | 17.3 (21.8) | 13.6 (14.8) | 23.6 (28.3) |
| **Educational level, %** |  |  |  |
| -Primary/secondary school | 42.9 | 41.4 | 30.8 |
| -High school | 23.8 | 17.2 | 33.3 |
| -University degree | 33.3 | 41.4 | 35.9 |
| **Mean BMI, Kg/m^2^ (SD)** | 27.3 (4.5) | 28.9 (2.9) | 27.7 (3.6) |
| **Smoking status:** |  |  |  |
| -Cigarette smoke (current or former), % | 66.7 | 72.4 | 74.4 |
| -Mean pack/year (SD) | 24.6 (21.5) | 28.1 (25.8) | 19.1 (20.0) |
| **Comorbidities, %** |  |  |  |
| -None | 4.8 | 10.3 | 2.6 |
| -GERD | 14.3 | 20.7 | 23.1 |
| -Cancer | 0.0 | 10.3 | 10.3 |
| -Respiratory diseases | 28.6 | 13.8 | 5.1 |
| -Cardiovascular diseases | 40.4 | 30.3 | 46.3 |
| -Others | 11.8 | 14.7 | 12.6 |
| **Familiarity for IPF, %** | 9.5 | 17.2 | 7.7 |
| **mMRC Breathlessness Scale grade^‡^, %** |  |  |  |
| -Grade 0 | 9.5 | 10.3 | 5.1 |
| -Grade 1 | 14.3 | 13.8 | 25.6 |
| -Grade 2 | 47.6 | 37.9 | 41.0 |
| -Grade 3 | 19.1 | 31.0 | 18.0 |
| -Grade 4 | 9.5 | 6.9 | 10.3 |
| **Exposed to occupational dust ≥10 years, %** | 42.9 | 55.7 | 53.9 |
| **Exposure to asbestos, %** | 33.3 | 37.9 | 28.2 |
| **Mean duration of asbestos exposure, years (SD)** | 7.6 (14.9) | 9.2 (15.3) | 7.0 (12.8) |
| **Mean WHODAS 2.0 score (SD)** | 23.9 (19.1) | 20.9 (19.0) | 22.3 (18.2) |
| **Mean HADS-A score (SD)** | 6.6 (4.2) | 6.1 (3.0) | 6.3 (3.1) |
| **Mean HADS-D score (SD)** | 6.5 (4.1) | 6.3 (3.9) | 6.8 (3.1) |
| **Pharmacological treatment, %** |  |  |  |
| - Pirfenidone | 47.6 | 55.2 | 64.1 |
| - Nintedanib | 52.4 | 44.8 | 35.9 |
| * Chi-squared test for categorical variables; Kruskal-Wallis test for normally-distributed and non normally distributed continuous variables, respectively. As all p-values were >0.05, they were not reported to avoid redundancy.  °Analysis restricted to 89 subjects in pharmacological treatment and with complete respiratory function tests at diagnosis and after 12 months of therapy.  SD= Standard deviation; FVC = Forced Vital Capacity; BMI= Body Mass Index; GERD= Gastro-esophageal reflux disease; mMRC= Medical Research Council; WHODAS 2.0= WHO Disability Assessment Schedule 2.0 tool; HADS-A= Hospital Anxiety and Depression Scale-Anxiety domain; HADS-D= Hospital Anxiety and Depression Scale-Depression domain.  ‡ Each grade of the MRC Breathlessness Scale was scored as follows: Grade 1 = Not troubled by breathlessness except on strenuous exercise; Grade 2 = Short of breath when hurrying on the level or walking up a slight hill; Grade 3 = Walks slower than most people on the level, stops after a mile or so, or stops after 15 minutes walking at own pace; Grade 4= Stops for breath after walking about 100 yards or after a few minutes on level ground; Grade 5 = Too breathless to leave the house, or breathless when undressing. | | | |
